# Optoacoustic inversion via convolution kernel reconstruction in the paraxial approximation and beyond

**DOI:** 10.1016/j.pacs.2018.10.004

**Published:** 2018-11-05

**Authors:** O. Melchert, M. Wollweber, B. Roth

**Affiliations:** Hannover Centre for Optical Technologies (HOT), Interdisciplinary Research Centre of the Leibniz Universität Hannover, Nienburger Str. 17, D-30167 Hannover, Germany

**Keywords:** Optoacoustics, Volterra integral equation of the second kind, Convolution kernel reconstruction, Tissue phantom

## Abstract

In this article we address the numeric inversion of optoacoustic signals to initial stress profiles. Therefore we study a Volterra integral equation of the second kind that describes the shape transformation of propagating stress waves in the paraxial approximation of the underlying wave-equation. Expanding the optoacoustic convolution kernel in terms of a Fourier-series, a best fit to a pair of observed near-field and far-field signals allows to obtain a sequence of expansion coefficients that describe a given “apparative” setup. The resulting effective kernel is used to solve the optoacoustic source reconstruction problem using a Picard-Lindelöf correction scheme. We verify the validity of the proposed inversion protocol for synthetic input signals and explore the feasibility of our approach to also account for the shape transformation of signals beyond the paraxial approximation including the inversion of experimental data stemming from measurements on melanin doped PVA hydrogel tissue phantoms.

## 1 Introduction

The *inverse* optoacoustic (OA) problem is concerned with the reconstruction of “internal” medium properties from “external” measurements of acoustic pressure signals. In contrast to the *direct* OA problem, referring to the calculation of a diffraction-transformed pressure signal at a desired field point for a given initial stress profile [1], [2], [3], [4], [5], [6], [7], one can distinguish two inverse OA problems: (I.1) the *source reconstruction problem*, where the aim is to invert measured OA signals to initial stress profiles upon knowledge of the mathematical model that mediates the underlying diffraction transformation [8], [6], [9], [10], [11], and, (I.2) a *kernel reconstruction problem*, where the task is to find a convolution kernel that accounts for the apparent diffraction transformation shown by the OA signal. The latter arises quite naturally in a paraxial approximation wherein both signals can be related via a Volterra integral equation of the second kind [12]. Note that while problem I.1 is well established in the field of optoacoustics, we here make a first attempt at solving problem I.2, i.e. the kernel reconstruction problem, and demonstrate how it can be utilized for the reconstruction of initial stress profiles from observed OA signals.

Owing to its immediate relevance for medical applications [13], [14], [15], [16], [17], [18], [19], current progress in the field of inverse optoacoustics is spearheaded by OA tomography (OAT) and imaging applications in line with (I.1) [20], [21], [22], [23], [24], [25], problem (I.2) has not yet received much attention. However, quite similar kernel reconstruction problems are well studied in the context of inverse-scattering problems in quantum mechanics [26], [27], [28], [29], and, from a more general point of view, are also studied in applied mathematics [30], [31], [32]. Note that analytic inversion formulae in OAT, aiming at reconstructing the full electromagnetic absorption distribution within the medium (see, e.g., Refs. [23], [24], [25]), assume that OA signals are detected from a full view, or, as in case of deconvolution reconstruction [33], still from a limited view of the object under scrutiny. If it is unfeasible to employ OAT techniques and one needs to resort to the inversion of data measured at a single point of the region of interest, due to either the inaccessibility of OAT inversion input or by other boundary conditions, kernel reconstruction in terms of (I.2) might provide an opportunity for OA inversion. However, note that the proposed approach does not evade the issue that reconstruction of data obtained from point detectors is, in general, not exact.

As a remedy, we here describe a numeric inversion scheme for problem (I.2), applicable to OA signals observed at a single field point, allowing to solve for a 1D absorption depth profile. Our aim is not to propose a competitive image reconstruction method for OAT applications that would require the reconstruction of full 3D domains from OA signals recorded at numerous detection angles. More precisely, in the presented article, we focus on the kernel reconstruction problem in the paraxial approximation to the optoacoustic wave-equation, where we suggest a Fourier-expansion approach to construct an approximate optoacoustic convolution kernel. We show that once (I.2) is solved for a given “apparative” setup, this then allows to subsequently solve (I.1) for different signals obtained using an identical apparative setup. A central and reasonable assumption of our approach is that the influence of the stress wave propagator on the shape change of the OA signal is negligible above a certain cut-off distance. After developing and testing the numerical procedure in the paraxial approximation, we assess how well the inversion protocol carries over to more prevalent optoacoustic problem instances, featuring the reconstruction for: (i) the full OA wave-equation, (ii) non Gaussian irradiation source profiles, and, (iii) measured signals exhibiting noise.

The article is organized as follows: in Section 2 we recap the theoretical framework of OA signal generation in the paraxial approximation, in Section 3 we discuss our approach to the OA kernel reconstruction problem, in Section 4 we illustrate the subsequent solution of the source reconstruction problem via the obtained approximate convolution kernel, and allude to the challenging problem of OA signal inversion beyond the paraxial approximation in Section 5. In Section 6, we conclude with a summary.

## 2 The direct OA problem in the paraxial approximation

The dominant microscopic mechanism contributing to the generation of acoustic stress waves is expansion due to photothermal heating [34]. In the remainder we assume a pulsed photothermal source with pulse duration short enough to ensure thermal and stress confinement [8]. Then, in case of a purely absorbing material exposed to an irradiation source profile with beam axis along the *z*-direction of an associated coordinate system, a Gaussian profile in the transverse coordinates ${\vec{r}}_{⊥}$ and nonzero depth dependent absorption coefficient *μ*_*a*_(*z*), limited to *z* ≥ 0 and varying only along the *z*-direction, the initial acoustic stress response to photothermal heating takes the form

(1) $$p_{0}(\vec{r})=\Gamma f_{0}\;\mu_{a}(z)\exp\left\{-|{\vec{r}}_{⊥}{|}^{2}/a_{B}^{2}-\int_{0}^{z}\mu_{a}(z^{\prime })\;dz^{\prime }\right\}.$$

Therein Γ, *f*_0_ and *a*_B_ signify the Grüneisen parameter, the intensity of the irradiation source along the beam axis and the 1/*e*-width of the beam profile orthogonal to the beam axis, respectively. Given the above initial instantaneous acoustic stress field $p_{0}(\vec{r})$, the scalar excess pressure field $p(\vec{r},t)$ at time *t* and field point $\vec{r}$ can be obtained by solving the inhomogeneous OA wave equation [4], [8]

(2) $$[\partial_{t}^{2}-c^{2}\Delta ]\;p(\vec{r},t)=p_{0}(\vec{r})\;\partial_{t}\;\delta (t),$$

with *c* denoting the sonic speed in homogeneous media. Putting the dispersion relation $\omega^{2}=c^{2}|\vec{k}{|}^{2}$ of a harmonic wave solution $p(\vec{r},t)=A\exp\{i(\omega t-\vec{k}\vec{r})\}$ with frequency *ω* and wave vector $\vec{k}=(k_{x},k_{y},k_{z})$ (satisfying the homogeneous wave equation) under scrutiny [35], it is possible to identify frequencies that correspond to solutions $p_{+}(\vec{r},t)$ and $p_{-}(\vec{r},t)$ that travel in positive and negative *z*-direction, respectively. This allows to derive a more simple propagation equation than Eq. (2) that forms an adequate approximation to *p*_+_, only. I.e., employing a first order expansion of the dispersion relation in the transverse parameter $\epsilon ={\left(k_{x}^{2}+k_{y}^{2}\right)}^{1/2}/|\vec{k}|$ yields a rational approximation $\omega_{+}^{2}-ck_{z}\omega_{+}-c^{2}(k_{x}^{2}+k_{y}^{2})/2=0$ that corresponds to a parabolic approximation of Eq. (2). Introducing time-retarded coordinates *t* → *τ* = *t* + *z*_D_/*c* the equivalent partial differential equation takes the form [4], [12], [35]

(3) $$[\partial_{\tau }\partial_{z}-(c/2)(\partial_{x}^{2}+\partial_{y}^{2})]\;p(\vec{r},t)=0.$$

Along propagation directions close to ${\vec{e}}_{z}$ a solution $p(\vec{r},t)$ to Eq. (3) yields a reasonable approximation to $p_{+}(\vec{r},t)$. In this paraxial approximation, the OA signal $p_{D}(\tau )\equiv p({\vec{r}}_{D},t)$ at a fixed field point ${\vec{r}}_{D}=(0,0,z_{D})$ along the beam axis can be related to the initial (*t* = 0) on-axis stress profile *p*_0_(*τ*) via a Volterra integral equation of the 2nd kind [12]

(4) $$p_{D}(\tau )=p_{0}(\tau )-\int_{-\infty }^{\tau }K(\tau -\tau^{\prime })\;p_{0}(\tau^{\prime })\;d\tau^{\prime }.$$

Therein the Volterra operator features a convolution kernel *K*(*τ* − *τ*′) = *ω*_D_ exp { − *ω*_D_(*τ* − *τ*′)}, mediating the diffraction transformation of the propagating stress waves [12]. A derivation of the exponential convolution kernel in terms of the transfer function method working in the spectral domain is detailed in Ref. [4]. The characteristic OA frequency $\omega_{D}=2c|z_{D}|/a_{B}^{2}$ effectively combines the defining parameters of the apparative setup **p**_sys_ ≡ (*c*, *a*_B_, *z*_D_). The acoustic near and far-field might be distinguished by means of the diffraction parameter $D=2|z_{D}|/(\mu_{a}a_{B}^{2})$, where near and far-field are characterized by *D* < 1 and *D* > 1, respectively. Subsequently we focus on OA signal detection in backward mode, i.e. *z*_D_ < 0.

**Fig. 1:**
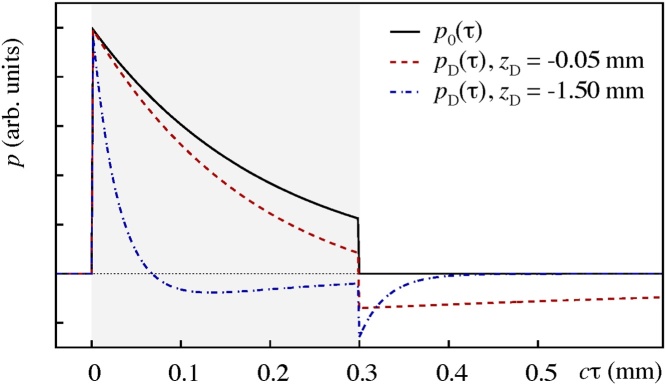
Illustration of OA signals obtained in the acoustic near-field (*D* = 0.22; red dashed curve) and far-field (*D* = 6.67; blue dash-dotted curve). The signals are obtained by solving the Volterra integral equation of the second kind, Eq. (4), that mediates the shape transformation of the initial acoustic stress profile (black solid curve) in the paraxial approximation. For the preparation of the initial stress profile it is assumed that *μ*_*a*_ = 5 mm^−1^ in the range [*cτ*_−_, *cτ*_+_] = [0, 0.3] mm and zero elsewhere. The convolution kernel reflects a Gaussian irradiation source profile with 1/*e*-width *a*_B_ = 0.3 mm.

As an exemplary application, Fig. (1) illustrates the solution of the direct OA problem, i.e. the forward calculation of OA signals via Eq. (4) for a problem setup that resembles the experimental setup reported in Ref. [36]. For a comparison of the prediction of OA signals in terms of the effectively 1D approach provided by Eq. (4) as opposed to the full 3D wave equation please refer to appendix B of Ref. [7].

## 3 Reconstruction of the OA convolution kernel

Note that the solution of the direct problem and inverse problem (I.1) in terms of Eq. (4) is feasible using standard numerical schemes based on, e.g., a trapezoidal approximation of the Volterra operator for a generic kernel [37], or highly efficient inversion schemes for the particular form of the above convolution kernel [11]. As pointed out earlier, considering inverse problem (I.2), we here suggest a Fourier-expansion of the convolution kernel involving a sequence of *N* expansion coefficients $\text{a}\equiv {\left\{a_{\ell }\right\}}_{0\leq \ell <N}$ and a cut-off distance *R* above which the resulting effective kernel is assumed to be zero, i.e.

(5) $$K(x;\text{a},R)=\sum_{\ell =0}^{N-1}a_{\ell }\;k_{\ell }(x;R)\;\Theta (R-x).$$

The expansion functions *k*_ℓ_(*x* ; *R*) are given by

(6) $$k_{\ell }(x;R)=\left\{\begin{array}{cc} 1, & \mathrm{if}\;\ell =0\\ \cos\left(2\pi \;\frac{\ell +1}{2}\frac{x}{R}\right), & \mathrm{if}\;\ell \;\mathrm{odd}\\ \sin\left(2\pi \;\frac{\ell }{2}\frac{x}{R}\right), & \mathrm{if}\;\ell \;\mathrm{even} \end{array}\right)$$

and Θ(·) signifies the Heavyside step-function. Then, for a suitable sequence **a**, the Fourier approximation to the Volterra integral equation, Eq. (4), reads

(7) $$p_{D}(\tau )=p_{0}(\tau )-\sum_{\ell =0}^{N-1}a_{\ell }\;\Phi_{\ell }(\tau ;R),$$

with auxiliary functions

(8) $$\Phi_{\ell }(\tau ;R)=\int_{-\infty }^{\tau }k_{\ell }(\tau -\tau^{\prime };R)\;\Theta (R-(\tau -\tau^{\prime }))\;p_{0}(\tau^{\prime })\;d\tau^{\prime }.$$

Now, consider a given set of input data (*p*_0_, *p*_D_) for known apparative parameters **p**_sys_, both in a discretized setting with constant mesh interval Δ, mesh points ${\left\{t_{i}\right\}}_{0\leq i\leq M}$ where *t*_0_ = 0, *t*_*i*_ = *t*_*i*−1_ + Δ, and *t*_*M*_ large enough to ensure a reasonable measurement depth. Then, bearing in mind that *τ*_*i*_ = *t*_*i*_ + *z*_D_/*c*, the optimal expansion coefficient sequence **a**^★^ can be obtained by minimizing the sum of the squared residuals (SSR)

(9) $$s(\text{a},R)=\sum_{i=0}^{M}{\left[\left(p_{0}(\tau_{i})-p_{D}(\tau_{i}))\;-\;\sum_{\ell =0}^{N-1}a_{\ell }\;\Phi_{\ell }(\tau_{i};R\right)\right]}^{2}.$$

In the above optimization formulation of inverse problem (I.2), we considered a trapezoidal rule to numerically evaluate the integrals that enter via the functions Φ_ℓ_(*τ*_*i*_ ; *R*). In an attempt to construct an effective Volterra convolution kernel *K*(*x* ; **a**, *R*) for a controlled setup with *a priori* known parameters **p**_sys_, one might use the high-precision “Gaussian-beam” estimator $a_{\ell }=(2\omega_{D}/R)\int_{0}^{R}k_{\ell }(x;R)\;\exp\{-\omega_{D}x\}\;dx$ to obtain an initial sequence **a**_0_ of expansion coefficients by means of which a least-squares routine for the minimization of Eq. (9) might be started. In a situation where, say, *a*_B_ is only known approximately or the assumption of a Gaussian beam profile is violated, one has to rely on a rather low-precision coefficient estimate obtained by roughly estimating the apparative parameters and resorting on the above “Gaussian-beam” estimate.

**Fig. 2:**
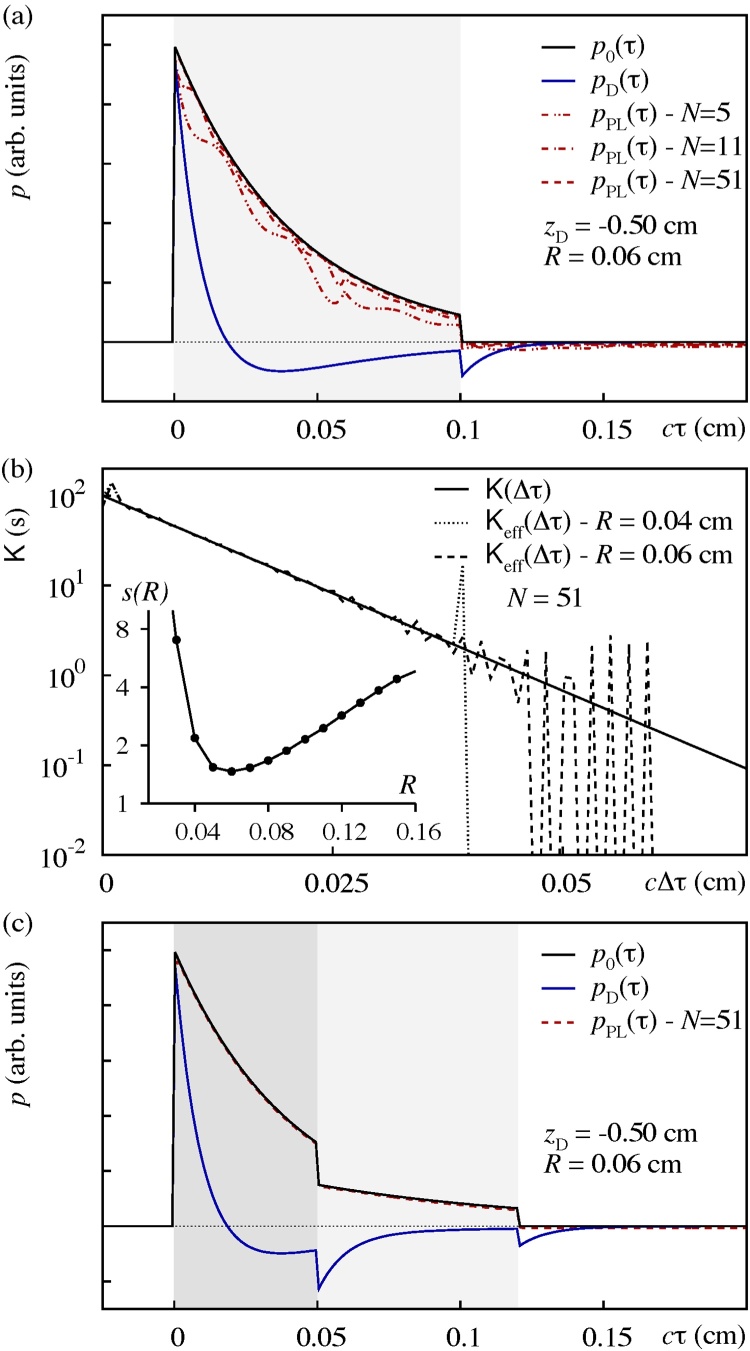
Kernel and source reconstruction within the paraxial approximation for system parameters **p**_sys_ = (*c*, *a*_B_, *z*_D_) ≡ (1 cm/*s*, 0.1 cm, − 0.5 cm). (a) Inversion input *p*_0_ (solid black line) and *p*_D_ (solid blue line) used to derive effective kernel for *N* = 5, 11, and 51 Fourier-coefficients and cut-off parameter *R* = 0.06 cm. Solution of the respective source reconstruction problems yields the estimates *p*_PL_ (dashed and dash-dotted red curves). (b) The main plot illustrates the effective kernel *K*_eff_(Δ*τ*) ≡ *K*(Δ*τ* ; **a**^★^, *R*) for two different cut-off distances *R* = 0.04 cm, and 0.06 cm. The inset shows the SSR *s*(*R*) ≡ *s*(**a**^★^, *R*) for *N* = 51 as function of the cut-off distance where the minimum is attained at *R* = 0.06 cm. (c) Solution *p*_PL_ of the source reconstruction problem for a OA signal *p*_D_ (solid blue line) resulting from a two-layer absorbing structure for the same system parameters as in (a). Source reconstruction is performed using the effective kernel for **p**_rec_ = (51, 0.06 cm) resulting from the gauge procedure.

An exemplary kernel reconstruction procedure is shown in Fig. 2 , where the OA signal *p*_D_ at **p**_sys_ = (1 cm/*s*, 0.1 cm, − 0.5 cm), i.e. *D* ≈ 3.75, is first obtained by solving the direct OA problem for Eq. (4) for an absorbing layer with *μ*_*a*_ = 24 cm^−1^ in the range *z* = 0 −0.1 cm, see black (*p*_0_) and blue (*p*_D_) curves in Fig. 2 (a). The set (*p*_0_, *p*_D_) is then used as inversion input to compute the effective Volterra kernel for various sets of reconstruction parameters **p**_rec_ = (*N*, *R*). In particular, considering *N* = 51, the minimal value of *s*(**a**^★^, *R*^★^) ≈ 1.47 is attained at *R*^★^ = 0.06 cm, see the inset of Fig. 2 (b). As evident from the main plot of Fig. 2 (b), the effective Volterra kernel for **p**_rec_ = (51, *R*^★^) follows the exact stress wave propagator for almost two orders of magnitude up to *c*Δ*τ* ≈ 0.05 cm. Beyond that limit, the noticeable deviation between both does not seem to affect the overall SSR *s*(**a**, *R*) too much. In this regard, note that the kernel approximated for the (non optimal) choice **p**_rec_ = (51, 0.04 cm) exhibits a worse SSR.

## 4 The inverse OA problem – source profile reconstruction

Note that the above Fourier-expansion approximation might be interpreted as a gauge procedure to adjust an effective Volterra kernel *K*(*x* ; **a**^★^, *R*) for an (possibly unknown) apparative setup **p**_sys_, here indirectly accessible through the diffraction transformation of the OA signal *p*_D_ relative to *p*_0_. That is, once the kernel reconstruction (I.2) is accomplished for a set of reference curves ${\left(p_{0},p_{D}\right)}_{\mathrm{ref}}$ under **p**_sys_, the source reconstruction problem (I.1) might subsequently be tackled also for all other OA signals measured under **p**_sys_ by solving the OA Volterra integral equation Eq. (4) in terms of a Picard-Lindelöf “correction” scheme [38]. The latter is based on the continued refinement of a putative solution, starting off from a properly guessed “predictor” $p_{\mathrm{PL}}^{\left(0\right)}(\tau )$, improved successively by solving

(10) $$p_{\mathrm{PL}}^{\left(n+1\right)}(\tau )=p_{D}(\tau )+\int_{-\infty }^{\tau }K(\tau -\tau^{\prime };{\text{a}}^{★},R)\;p_{\mathrm{PL}}^{\left(n\right)}(\tau^{\prime })\;d\tau^{\prime }.$$

From a practical point of view we terminated the iterative correction scheme as soon as the max-norm $c_{n}\equiv ∥p_{\mathrm{PL}}^{\left(n+1\right)}(\tau )-p_{\mathrm{PL}}^{\left(n\right)}(\tau )∥$ of two successive solutions decreases below *c*_*n*_ ≤ 10^−6^. We here refer to the final estimate simply as *p*_PL_. Note that, attempting a solution of (I.1) in the acoustic near-field, a high-precision predictor can be obtained by using the initial guess $p_{\mathrm{PL}}^{\left(0\right)}\equiv p_{D}$. This is a reasonable choice since one might expect the change of the OA near-field signal due to diffraction to be still quite small. Further, source reconstruction in the acoustic far-field might be started using a high-precision predictor obtained by integrating the OA signal *p*_D_ in the far-field approximation [11]. In contrast to this, low-precision predictors for both cases can be obtained by setting $p_{\mathrm{PL}}^{\left(0\right)}\equiv c_{0}$, where, e.g., *c*_0_ = 0.

The solution of the source reconstruction problem for the OA signal *p*_D_ used in the approximation of the Volterra kernel for the above setting **p**_sys_ = (1 cm/*s*, 0.1 cm, − 0.5 cm) is shown in Fig. 2 (a). We assessed the scaling of the speed of convergence, measured using the number of steps *n*_max_ taken by the Picard-Lindelöf correction scheme, with increasing number of expansion coefficients *N*, finding *n*_max_ ∝ *N*^1.3^. Note, however, that the computational burden of the Picard-Lindelöf correction scheme is inferior to the minimization of the SSR according to Eq. (9). The apparent agreement of the data curves *p*_PL_ for **p**_rec_ = (51, *R*^★^) and *p*_0_ does not come as a surprise since *p*_D_ was used for the gauge procedure in the first place. As a remedy we attempt a source reconstruction for a second independent OA signal, simulated for the same apparative setting only with two absorbing layers *μ*_*a*,1_ = 24 cm^−1^ from *z* = 0 −0.05 cm and *μ*_*a*,2_ = 12 cm^−1^ from *z* = 0.05 − 0.12 cm. As evident from Fig. 2 (c), inversion using the effective Volterra kernel from the previous gauge procedure yields a reconstructed stress profile *p*_PL_ in excellent agreement with the underlying exact initial stress profile *p*_0_.

## 5 Inversion beyond the paraxial approximation

**Fig. 3:**
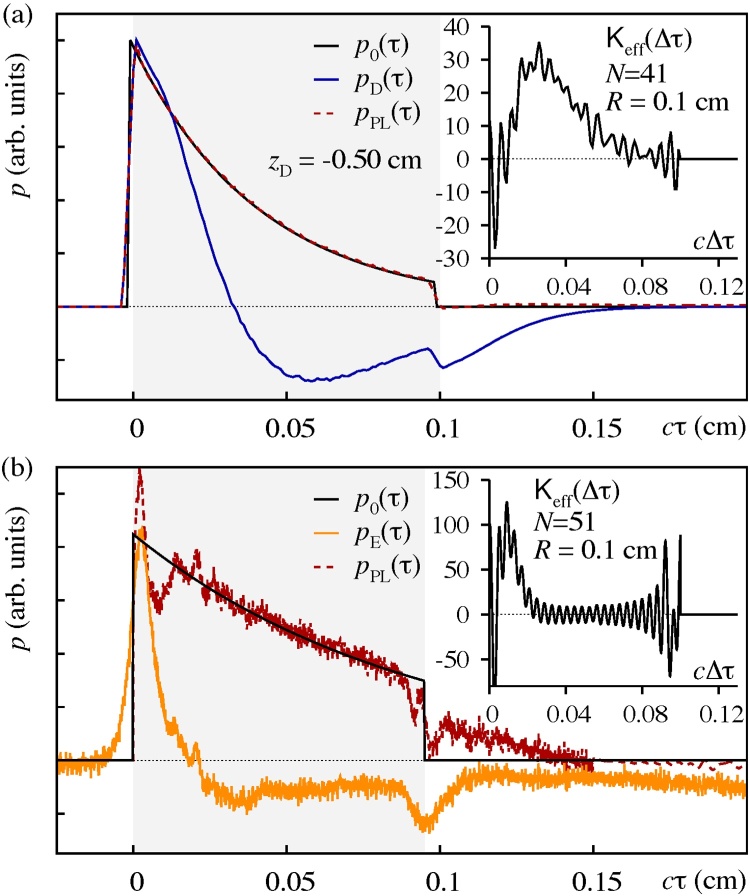
Inversion of OA signals to initial stress profiles beyond the paraxial approximation. Both figures illustrate the kernel and source reconstruction procedures for (a) inversion of an OA signal featuring a top-hat irradiation source profile (see text). The main plot shows the input (*p*_0_, *p*_D_) to the inversion procedure (solid black and blue lines, respectively) as well as the reconstructed initial stress profile *p*_PL_ (dashed red line), and, (b) inversion of an OA signal resulting from an actual measurement [10]. The main plot shows the synthetic initial stress profile *p*_0_ (solid black line) used during the gauge procedure as well as the inversion input *p*_E_ (orange line) for which the reconstructed initial stress profile *p*_PL_ (dashed red line) is obtained. In both figures, the inset illustrates the effective Volterra kernel resulting from the Fourier-approximation.

Given the apparent feasibility of the kernel reconstruction routine as a gauge procedure to model the diffraction transformation of OA signals in terms of an effective stress wave propagator in the framework of the OA Volterra integral equation, we next address the inversion of OA signals to initial stress profiles beyond the paraxial approximation. Therefore, we first consider a borderline far-field signal for a top-hat irradiation source

(11) $$f({\vec{r}}_{⊥})=\left\{\begin{array}{cc} 1, & \mathrm{if}\;|{\vec{r}}_{⊥}|\leq \rho_{0}\\ \exp\{-{\left(|{\vec{r}}_{⊥}|-\rho_{0}\right)}^{2}/a_{B}^{2}\}, & \mathrm{if}\;|{\vec{r}}_{⊥}|>\rho_{0} \end{array}\right),$$

recorded at the system parameters **p**_sys_ = (*c*, *ρ*_0_, *a*_B_, *z*_D_) = (1 cm/*s*, 0.1 cm, 0.1 cm, − 0.50 cm), and thus *D* = 2|*z*_D_|/(*μ*_a_(*a*_B_ + *ρ*_0_)) ≈ 1.04, obtained via an independent forward solver for the full OA wave equation designed for the solution of the OA Poisson integral for layered media [8], [10]. The inversion results are summarized in Fig. 3 (a), where the kernel reconstruction (inset) and source reconstruction (main plot) are shown for the parameter set **p**_rec_ = (41, *R* = 0.1 cm). The position of the peak of the effective kernel seems due to the finite extension *ρ*_0_ of the employed top-hat beam profile (bear in mind that originally, the underlying 1D approach assumes a Gaussian beam profile). We find that, the larger the respective top-hat width *ρ*_0_, the further out that peak occurs. Consequently, the cut-off distance *R* above which the kernel is assumed to vanish needs to be chosen large enough to still enclose the peak of the kernel. The excellent agreement of the stress profiles *p*_0_ and *p*_PL_ suggests that the kernel reconstruction routine also applies to a more general OA setting, based on the full OA wave equation.

Finally, we consider an OA signal resulting from an actual measurement on PVA hydrogel based tissue phantoms [10]. In this case we carefully estimated the apparative parameters **p**_sys_ = (150000 cm/*s*, 0.054 cm, 0.081cm/*s*, − 0.3 cm) as well as *μ*_*a*_ = 11 cm^−1^ in the range *z* = 0 −0.095 cm, i.e. *D* ≈ 6.73, in order to create a set of synthetic input data by means of which an appropriate kernel gauge procedure can be carried out. The result of the procedure using **p**_rec_ = (51, 0.1 cm) is shown in Fig. 3 (b). So as to perform the source reconstruction for the experimental signal *p*_E_, we considered data within the interval *cτ* = [0, 0.15] cm, only. As evident from the figure, the reconstructed stress profile *p*_PL_ fits the signal *p*_0_ used in the gauge procedure remarkably well.

## 6 Conclusions

In the presented article we have introduced and discussed the kernel reconstruction problem in the paraxial approximation of the optoacoustic wave equation for both, synthetic input data and experimental data resulting from controlled measurements on melanin doped PVA hydrogel tissue phantoms. We suggested a Fourier-expansion approach to approximate the convolution kernel which takes a central role in the theoretical framework. The developed approach proved useful as gauge procedure by means of which the diffraction transformation experienced by OA signals can effectively be modeled, allowing to subsequently solve the source reconstruction problem in the underlying apparative setting. From this numerical study we found that the developed approach extends beyond the framework of the paraxial approximation and also allows for the inversion of OA signals described by the full OA wave equation in the acoustic far field. It would be tempting to explore other kernel expansions in terms of generalized Fourier series and to assess the presented method with regard to different signal-to-noise ratios in the input data. Also, the effects of acoustic attenuation, the impulse response of the employed transducer and uncertainty in the system parameters might be studied in detail so as to probe the limits of the proposed inversion scheme. Such investigations are currently in progress with the aim to shed some more light on this intriguing inverse problem in the field of optoacoustics and to facilitate a complementary approach to conventional OA signal inversion.

A Python implementation of our research-code for the solution of inverse problems (I.1) and (I.2), along with all scripts needed to reproduce the figures is publicly available on one of the authors figshare profile, see Ref. [39].

Finally, note that here we discussed the problem of optoacoustic inversion in the limit of unscattered transmission. However, in general, the propagation of light in biological tissue is goverened by a scattering coefficient *μ*_*s*_ with finite value [40]. In the limit *μ*_*s*_ ≫ *μ*_*a*_ this renders the transmission process effectively diffusive. As a consequence, the light beam may behave as Gaussian only for depths >1 mm. In this depth range, optical-resolution photoacoustic microscopy (OR-PAM) [41] might be of interest.

## Conflict of interests

None.
